# Maternal exposure to polycyclic aromatic hydrocarbons in South Texas, evaluation of silicone wristbands as personal passive samplers

**DOI:** 10.1038/s41370-021-00348-y

**Published:** 2021-06-15

**Authors:** Itza Mendoza-Sanchez, Inyang Uwak, Louise Myatt, Allison Van Cleve, Jairus C. Pulczinski, Kristal A. Rychlik, Stephen Sweet, Tara Ramani, Josias Zietsman, Misti Levy Zamora, Kirsten Koehler, Genny Carrillo, Natalie M. Johnson

**Affiliations:** 1 Department of Environmental and Occupational Health, Texas A&M University School of Public Health, College Station, TX USA; 2 Department of Environmental Health and Engineering, Johns Hopkins Bloomberg School of Public Health, Baltimore, MD USA; 3 Geochemical and Environmental Research Group, Texas A&M University, College Station, TX USA; 4 Environment and Air Quality Division, Texas A&M Transportation Institute, College Station, TX USA

**Keywords:** Polycyclic aromatic hydrocarbons, Air pollution, Early life exposure, Inhalation exposure, Vulnerable populations

## Abstract

**Background:**

Prenatal exposure to polycyclic aromatic hydrocarbons (PAHs) is associated with adverse health effects in children. Valid exposure assessment methods with accurate spatial and temporal resolution across pregnancy is a critical need for advancing environmental health studies.

**Objective:**

The objective of this study was to quantify maternal PAH exposure in pregnant women residing in McAllen, Texas where the prematurity rate and childhood asthma prevalence rates are high. A secondary objective was to compare PAH levels in silicone wristbands deployed as passive samplers with concentrations measured using standardized active air-sampling techniques.

**Methods:**

Participants carried a backpack that contained air-sampling equipment (i.e., filter and XAD sorbent) and a silicone wristband (i.e., passive sampler) for three nonconsecutive 24-h periods. Filters, XAD tubes, and wristbands were analyzed for PAHs.

**Results:**

The median level of exposure for the sum of 16 PAHs measured via active sampling over 24 h was 5.54 ng/m^3^ (filters) and 43.82 ng/m^3^ (XADs). The median level measured in wristbands (WB) was 586.82 ng/band. Concentrations of the PAH compounds varied across sampling matrix type. Phenanthrene and fluorene were consistently measured for all participants and in all matrix types. Eight additional volatile PAHs were measured in XADs and WBs; the median level of exposure for the sum of these eight PAHs was 342.98 ng/m^3^ (XADs) and 632.27 ng/band. The silicone wristbands (WB) and XAD sorbents bound 1-methynaphthalyne, 2-methylnaphthalene, biphenyl following similar patterns of detection.

**Significance:**

Since prior studies indicate linkages between PAH exposure and adverse health outcomes in children at the PAH levels detected in our study, further investigation on the associated health effects is needed. Data reflect the ability of silicone wristbands to bind smaller molecular weight, semivolatile PAHs similar to XAD resin. Application of wristbands as passive samplers may be useful in studies evaluating semivolatile PAHs.

## Introduction

Polycyclic aromatic hydrocarbons (PAHs) represent a complex mixture of organic compounds with two or more fused aromatic rings formed as the result of incomplete combustion. PAHs are ubiquitous environmental contaminants due to their multiple sources, including vehicle emissions, tobacco smoke, cooking, and other combustion processes [1]. PAHs with lower volatility are bound to particulate matter (PM), including the fine PM, particles with a diameter smaller than 2.5 μm (PM_2.5_) and ultrafine fractions, which represent nano-sized particles <0.1 μm in diameter [2].

PAHs are known reproductive/developmental toxicants that can cross the placenta and exert effects on the developing fetus [3]. Maternal exposure to PAHs during pregnancy is associated with adverse effects on children’s health. Occupational exposure during gestation has been linked with increased risk of small for gestational age infants [4] and birth defects [5, 6]. In a multiethnic longitudinal cohort in New York City, prenatal exposure to ambient PAHs was associated with fetal growth reduction, adverse cognitive development, and obesity in childhood [7–10]. Similarly, in an analogous cohort study of Polish mothers living in Krakow, investigators observed associations between prenatal PAH exposure and adverse cognitive development and respiratory outcomes, including wheeze and decreased lung function [11–14]. Notably, exposure to PAHs showed a greater impact on infant birthweight reduction in comparison to assessment only considering PM_2.5_ exposure, highlighting the importance of chemical characterization of PM in epidemiologic studies [15].

We carried out a pilot study in Hidalgo County, Texas, near the U.S.–Mexico border to characterize personal exposure to PM_2.5_, black carbon (BC), and nicotine in pregnant women [16]. Our rationale was based on the heightened prevalence of childhood asthma in this region of Texas [17]. Hidalgo County also exhibits a higher prematurity rate (14.8%) compared to the state (12.9%), a birth outcome often observed in regions with elevated PM pollution [18]. The objective of this study was to quantify maternal PAH exposure in pregnant women residing in the McAllen–Edinburg–Mission region, the most populated area in Hidalgo County. A secondary objective was to compare PAH levels in silicone wristbands deployed as passive samplers with concentrations measured using standardized active air-sampling techniques. Previous studies have used wristbands worn by participants to obtain integrated inhalation and dermal exposures of PAH and other organic compounds [19–21]. Our objective differs from those studies in which we are interested in estimating PAH air concentrations from values obtained in passive samplers. To obtain those estimates, recent evidence supports the utility of obtaining wristband–air partition coefficients [22–24]. Our secondary objective contributes to existing literature addressing the potential of silicone wristbands to yield concentrations of organic compounds in air comparable to active sampling.

## Methods

### Study population

Participants (*n* = 17) were recruited from Rio Grande Regional Women’s Clinics located within the McAllen–Edinburg–Mission region. Inclusion criteria included healthy (non-asthmatic and nondiabetic) women, 21–35 years of age, with singleton pregnancies in their third trimester, residing in a nonsmoking household. All study procedures were approved by Texas A&M University Institutional Review Board, and participants were provided written informed consent in either English or Spanish based on participant choice.

### Study design

Sampling occurred between June 2015 and April 2016. Each participant was asked to carry monitoring equipment for a duration of 24 h, prior to their scheduled prenatal care appointment. In order to investigate individual day-to-day variation in exposure, we used a longitudinal sampling design entailing three nonconsecutive 24-h measurements, every 2 weeks (within a 6-week period), which coincided with prenatal care visits occurring every other week. The day prior to scheduled prenatal care visits, community health workers delivered a light-weight backpack containing air-sampling equipment with a precleaned silicone wristband attached near the inlet mounted on the backpack’s shoulder strap (Fig. S1). Participants were instructed to carry the backpack with them at all times and place within their breathing zone during sleep/rest. Participants returned the monitoring equipment (~24 h later) during the clinic visit. Total sampling time was recorded, and the active sampling pump was turned off. PTFE (polytetrafluoroethylene) filters and XAD tubes (described in “Active and passive air sampling”) were removed using sterile forceps and placed in clean petri dishes, sealed or covered with foil, and placed into PTFE bags, respectively. Likewise, wristbands were covered with foil and placed into PTFE bags to block light and any further chemical sorption. All sample matrices were stored at −20 °C until transport to Texas A&M University. Clinic staff also collected a small hair sample from the base of the neck to test for cotinine, a metabolite of nicotine, which was carried out at Johns Hopkins University [16]. This was done to rule out exposure to environmental tobacco smoke, which is a common source of PAHs.

### Active and passive air sampling

A personal environmental monitor (PEM, MSP Inc.) was used as a single-stage impactor PM_2.5_ inlet at an overall flow rate of 5 L min^−1^ by an external pump (BGI 400, Mesa Labs, Inc.) downstream a personal DataRAM^TM^ (pDR-1200, Thermo Scientific Corp., Waltham, Mass.) Temperature and relative humidity were tracked using a HOBO Temperature and Humidity Data Logger (Onset Computer Corporation, Pocasset, MA, USA). A 37-mm Teflon filter was used for PM_2.5_ analysis and gravimetric calibration. Details on maternal exposure to PM_2.5_, BC, and nicotine are reported separately [16]. To measure PAH concentrations, a second line from the pump was used to draw air through a 2-μm pore size, 37-mm PTFE filter (Pall Corporation, Ann Arbor, MI) followed by an XAD-2 sorbent tube (SKC, Inc., Eighty Four, PA) at a flow rate of 1 L min^−1^. Silicone bracelets (i.e., wristbands) were purchased in bulk from 24hourwristbands.com (Houston, TX). Average individual wristband weight (2.3 g) was measured in the laboratory, and a volume of 2 ml was obtained by displacement. A precleaned silicone wristband was attached near the inlet of the active air sampler. Previous studies have shown that when participants wear wristbands on their wrist, concentrations measured represent inhalation and dermal exposures [19–21, 25]. Unlike previous studies, we placed bands on the backpack strap to be able to achieve our secondary objective of comparing passive versus active sampling and calculating wristband to air partition coefficients. This setting allowed us to control for other variables that could affect air–wristband chemical uptake, such as dermal–wristband interactions, showering, use of personal care products among others.

### Filter/XAD extraction and wristband pre-cleaning/post-deployment extraction

PTFE filters and XAD resin tubes were placed into vials, and 100 μL of PAH surrogate mix was added to the samples. Samples were then Soxhlet-extracted with dichloromethane for 16 h. Extracts were concentrated to a final volume of 1 ml and spiked with a PAH internal standard mix. Silicone wristbands were precleaned and extracted according to previous methods [26]. To reduce any background contamination, wristbands (13 at a time) were washed in 1:1 ethyl acetate:hexane (three times) followed by 1:1 ethyl acetate:methanol (twice) by shaking at 60 rpm for 2.5 h. After the final wash, the bands were dried under high purity nitrogen, wrapped in foil, and stored in airtight PTFE bags until deployment. Post-deployment, prior to extraction, we gently rinsed the silicone wristband with deionized water to remove any surface particulates and then briefly rinsed with isopropanol to dry any residual water. Next, wristbands were extracted twice in 100-mL ethyl acetate by shaking for 2 h at 60 rpm. PAH surrogates were added to each sample at the start of extraction. After orbital shaking, the two extract fractions were combined and reduced to 1 mL and analyzed at the same time as filters and XAD tubes at the Geochemical and Environmental Research Group at Texas A&M University.

### PAH analysis

Filters, XAD tubes, and wristbands were analyzed for the 16 EPA-priority PAHs, except that we did not quantify naphthalene and instead measured benzo(e)pyrene. Thus, this set included acenaphthylene, acenaphthene, fluorene, phenanthrene, anthracene, fluoranthene, pyrene, benzo(a)anthracene, chrysene, benzo(k)fluoranthene, benzo(a)pyrene, dibenz(a,h)anthracene, benzo(b)fluoranthene, benzo(e)pyrene, indeno[1,2,3-c,d]pyrene, and benzo(g,h,i)perylene. In addition, we measured eight volatile PAHs in the XAD and wristband extracts including 1-methylnaphthalene, 2-methylnaphthalene, biphenyl, 2,6-dimethylnaphthalene, 1,6,7-trimethylnaphthalene, 1-methylphenanthrene, dibenzothiophene, and perylene. PAHs were analyzed using a Hewlett-Packard 6890 gas chromatograph (GC) coupled with a Hewlett-Packard 5973 mass selective detector. Separation of PAHs was accomplished with a DB-5 MS fused silica capillary column (30 m × 0.25-mm i.d., 0.50-μm film thickness, Agilent Technologies). The GC oven was temperature programmed to increase from an initial temperature of 60–150 °C at 15 °C per min, then at 5 °C per min to 220 °C, and ramped at 10 °C per min to a final temperature of 300 °C with a final holding time of 10 min. PAH compound identification was based on the comparison of the retention time and mass spectra of selected ions with the calibration standards. The target compounds were quantified using their relative response factors to the appropriate surrogate standards (d10-naphthalene, d10-acenaphthene, d10-phenanthrene, and d12-chrysene), which were calculated using a 5-point calibration analyzed at the beginning of each sequence. The limits of quantification ranged across matrix and PAH, e.g., from 0.01 to 0.38 ng/m^3^ for filter samples, from 0.01 to 10.93 ng/m^3^ for XAD samples, and from 0.03 to 56.85 ng/wristband. Recoveries of surrogate standards added prior to sample extraction ranged from 68.7 to 90.1%. Instrumental calibrations were checked by injection of the continuing calibration solution. The GC/MS calibration was verified before, during, and after each analytical sequence, and the calibration check was maintained within ±15% for all analytes of interest. Quality control samples, such as field and instruments blanks, were included in daily analysis. Concentrations in the instrument blanks were below the method detection limit. The field blanks included filters, XADs, and silicone wristbands deployed in the field during the time of sampling but kept under inert conditions. Field blanks were processed identically to participant samples. Average detection across compounds ranged from 0.10 to 0.77 ng/m^3^ for filter samples, from 0.00 to 10.22 ng/m^3^ for XAD samples, and from 1.07 to 247.64 ng/wristband. In the case of detection, average values field blanks were subtracted from the final participant concentrations for each analyte.

### Statistical analysis

The amount of PAH compounds bound to wristband was reported by mass (ng) in the wristband over the 24-h sampling period. Concentrations of PAHs in air were calculated dividing the mass of PAH (ng) bound to XADs and filters by volume of air calculated from flow rate and time. True missing values, as a result from non-collections in the field, were not included. Concentrations below the limit of detection were assigned half of the lowest detectable level measured in the samples. Summary statistics of the central tendency and spread of concentrations were determined for individual PAH compounds for each participant and each round. To evaluate the potential impact of temperature on PAH distributions, we calculated the average daily temperature and relative humidity for each sampling day and conducted a Spearman correlation analysis between PAHs in different media with temperature. Differences of statistical significance among measurements aggregated in different groups were evaluated using the Kruskall–Wallis and Wilcoxon signed-rank test. The groups included in the analysis of differences are: all 24 PAHs measured for each participant grouped by round, and all 17 participants’ PAH measured for each PAH grouped by round. Standardization and clustering were conducted followed by heatmaps to visually identify outstanding individuals in terms of PAH concentrations and main PAH profiles for XAD and WBs. Statistical analysis was conducted using R (version 3.5.3) with different packages installed.

## Results

Using a community-based approach, we recruited 17 participants to carry air monitoring equipment for three separate 24-h periods over a 6-week period. Only one participant did not complete all three sampling days, which resulted in a total of 50 sampling days. Our research team trained community health workers to deliver the air-sampling equipment to participant homes. This reduced the burden on the study participant and allowed for daily activities to continue as usual. In some instances, this design, including the home drop-off and clinic return of equipment resulted in some of the matrices (filters, XADs or WBs) to be categorized as non-collections in the field. This included 8/50 filters (16%), 3/50 XADs (6%), and 7/50 WBs (14%). Thus, we were able to collect and measure the majority of returned participant samples, ranging from 84 to 94%.

### Overall concentrations of PAH compounds in filters, XADs, and wristbands

In order to gain an overview of PAH concentrations in the different matrices, we combined the data from all participants for all three rounds and calculated the central tendency and spread of individual PAH concentrations. Among the 16 priority PAHs (Table 1), phenanthrene has the highest average concentrations in filters (23.73 ng/m^3^) and WBs (362.87 ng/band), while fluorene had the highest average concentration in the XADs (22.93 ng/m^3^). For the additional eight PAHs measured in XADs and WBs (Table 2), biphenyl has the highest average concentrations in both the XADs (95.46 ng/m^3^) and WBs (260.40 ng/band). We observed a low degree of influence of temperature on PAH measured in XAD matrix (see Table S1 with Spearman correlation results) but no influence on WB or filters.

**Table 1:** Concentrations of 16 criteria PAHs in filters, XADs, and wristbands, shown as mean ± standard deviation (median).

	Log (koa)	Molecular weight	Filter (ng/m^3^)	XAD (ng/m^3^)	Wristband (ng/band)
Acenaphthylene	6.3	152.2	2.43 ± 14.61 (0.10)	9.09 ± 16.70 (5.27)	9.39 ± 15.01 (6.80)
Acenaphthene	6.0	154.2	3.91 ± 22.74 (0.36)	14.49 ± 10.91 (11.17)	23.16 ± 45.86 (12.78)
Fluorene	6.6	166.2	6.05 ± 36.69 (0.33)	22.93 ± 31.12 (11.38)	63.47 ± 83.04 (44.22)
Phenanthrene	7.6	178.2	23.73 ± 96.33 (1.00)	14.17 ± 18.26 (6.70)	362.87 ± 195.53 (385.85)
Anthracene	7.1	178.2	5.59 ± 24.70 (0.09)	12.99 ± 78.18 (1.01)	22.93 ± 51.42 (9.01)
Fluoranthene	8.6	202.3	3.76 ± 16.19 (0.24)	1.62 ± 4.20 (0.80)	13.42 ± 19.49 (10.17)
Pyrene	8.2	202.3	5.05 ± 22.40 (0.33)	1.48 ± 3.82 (0.62)	47.85 ± 85.63 (20.21)
Benzo(a)anthracene	9.9	228.3	2.01 ± 8.20 (0.39)	4.38 ± 2.21 (3.59)	7.76 ± 19.26 (4.76)
Chrysene	10.2	228.3	2.21 ± 10.60 (0.28)	1.72 ± 3.58 (0.44)	25.62 ± 27.62 (25.84)
Benzo(k)fluoranthene	11.4	252.3	1.68 ± 9.16 (0.17)	0.20 ± 0.53 (0.04)	3.63 ± 3.46 (2.71)
Benzo(a)pyrene	11.2	252.3	2.19 ± 11.43 (0.25)	0.35 ± 0.48 (0.13)	1.00 ± 1.54 (0.43)
Dibenz(a,h)anthracene	12.7	278.4	3.86 ± 23.67 (0.10)	0.62 ± 1.36 (0.12)	1.68 ± 4.56 (0.40)
Benzo(b)fluoranthene	11.3	252.3	1.87 ± 9.57 (0.27)	0.22 ± 0.47 (0.04)	2.33 ± 6.98 (0.52)
Benzo(e)pyrene	11.2	252.3	1.85 ± 8.84 (0.23)	0.37 ± 0.82 (0.12)	0.93 ± 3.36 (0.09)
Indeno[1,2,3-cd]pyrene	12.4	276.3	2.69 ± 14.13 (0.20)	0.39 ± 0.68 (0.15)	0.24 ± 0.25 (0.14)
Benzo(g,h,i)perylene	12.6	276.3	3.84 ± 16.09 (0.48)	0.16 ± 0.33 (0.06)	0.54 ± 1.10 (0.15)
Σ16 PAHs			72.73 ± 327.57 (5.54)	85.19 ± 113.83 (43.82)	586.82 ± 434.42 (528.24)

**Table 2:** Concentrations of 8 volatile PAHs in XADs and wristbands, shown as mean ± standard deviation (median).

	Log (koa)	Molecular weight	XAD (ng/m^3^)	Wristband (ng/band)
1-Methylnaphthalene	5.5	142.2	57.26 ± 75.07 (25.46)	139.93 ± 436.01 (43.27)
2-Methylnaphthalene	5.5	142.2	93.50 ± 134.65 (34.38)	174.78 ± 413.21 (85.18)
Biphenyl	6.5	154.2	95.46 ± 72.20 (92.16)	260.40 ± 256.56 (248.15)
2,6-Dimethylnaphthalene	6.6	156.2	73.28 ± 83.83 (48.21)	23.14 ± 23.60 (15.91)
1,6,7-Trimethylnaphthalene	7.3	184.3	16.03 ± 31.53 (4.83)	13.23 ± 18.33 (6.30)
1-Methylphenanthrene	7.8	190.0	4.94 ± 21.41 (1.35)	139.93 ± 436.01 (43.27)
Dibenzothiophene	8.0	184.3	1.64 ± 2.85 (0.78)	4.21 ± 3.35 (2.85)
Perylene	11.4	252.3	0.88 ± 1.24 (0.60)	2.08 ± 1.92 (2.03)
∑ 8 PAHs			342.98 ± 250.25 (269.23)	632.27 ± 1078.04 (403.38)

### Distribution and statistical significant differences in the three media grouped by rounds

**Fig. 1:**
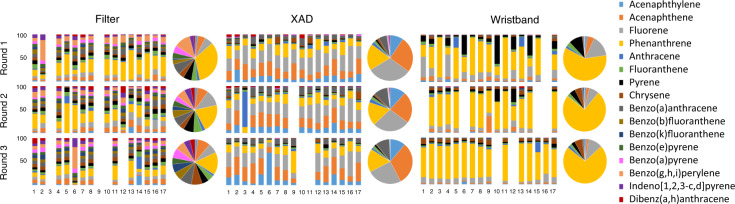
Percentage contribution of 16 EPA PAHs in individual’s filters, XADs, and WBs grouped by sampling round. Pie charts are percentages of PAHs medians of all individuals. PAHs are listed in order of molecular weight, which is related to volatility.

**Fig. 2:**
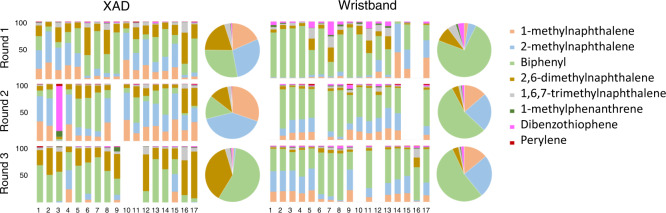
Percentage contribution of eight volatile PAHs in individual’s XAD and WB grouped by sampling round. Pie charts are percentages of PAHs medians of all individuals.

Figure 1 shows the percent contribution of PAH compounds to participants’ filters, XADs, and WBs for the 16 priority PAHs analyzed. In general, PAHs profiles were different for the three media, as expected. Filter profiles were composed of small percentages of several PAH compounds, while XAD and WB profiles were mainly composed of a few predominant PAH compounds. Phenanthrene and fluorene concentrations were consistently measured in the three rounds, across all three media, contributing up to 33% and 12% in filters, 19% and 31% in XADs, and 74% and 16% in WBs, respectively. In all the rounds, acenaphthene contributed 26% to XADs and 7% to filters on average, acenaphthylene was consistently measured in the XAD matrix at about 16% contribution, and benzo(g,h,i)perylene concentrations contributions in filter samples were on average 12%. Figure 2 shows the individual PAH compound contribution in XAD and WB samples for the eight additional volatile PAHs analyzed. Out of the eight PAHs, the first four with low molecular weight (1-methynaphthalyne, 2-methylnaphthalene, biphenyl, and 2,6-dimethylnaphthalene) represented nearly 90% of PAHs concentrations in the WBs and XADs. The main differences observed between XAD and WB are that their PAH profiles are different in all three rounds. For example, 1-methylnapthalene represents <10% in WB and around 25% in XAD for round 1, while the opposite is observed in round 3 (higher percentage in WB vs. XAD).

High variation in PAH concentrations was observed between the three rounds. Results of statistically significant differences evaluated for PAHs measured (Table S2) showed that acenapthene, chrysene, and dibenz(a,h)anthracene were different when grouped by round (Wilcoxon signed-rank *p* < 0.05) across all media (filter, XAD, and WB). Most PAHs showed statistically significant differences across media and between rounds except for biphenyl, acenaphthylene, benzo(g,h,i)perylene, 2,6-dimethylnaphthalene, and 1,6,7-trimethylnaphthalene. Concentrations were also aggregated by PAH’s measured for each participant and compared among rounds. Results (Table S3) showed that there are four participants with distinct PAH profiles between rounds for concentrations measured in XAD (1, 2, 3, and 8), one participant for concentrations measured in WB [17] and nine participants in filters (1, 2, 4, 6, 7, 8, 13, and 16).

### Concentration patterns of PAH among participants and media

We used heatmaps to visually identify single individuals with high concentrations in each round and groups of individuals with similar patterns of PAH concentrations. The data in the heatmap were standardized by subtracting the PAH compound’s mean to each PAH measured and dividing the result by the PAH compound’s standard deviation. Thus, the scale bar in the heatmaps represents the distance away from the average; positive numbers indicate concentrations above the average and negative numbers represent concentrations below the average. Heatmaps with participants as the main variable (Figs. S2 and S3), is helpful to identify specific participants in each round with high concentrations of PAHs in filter, XAD, and WB.

**Fig. 3:**
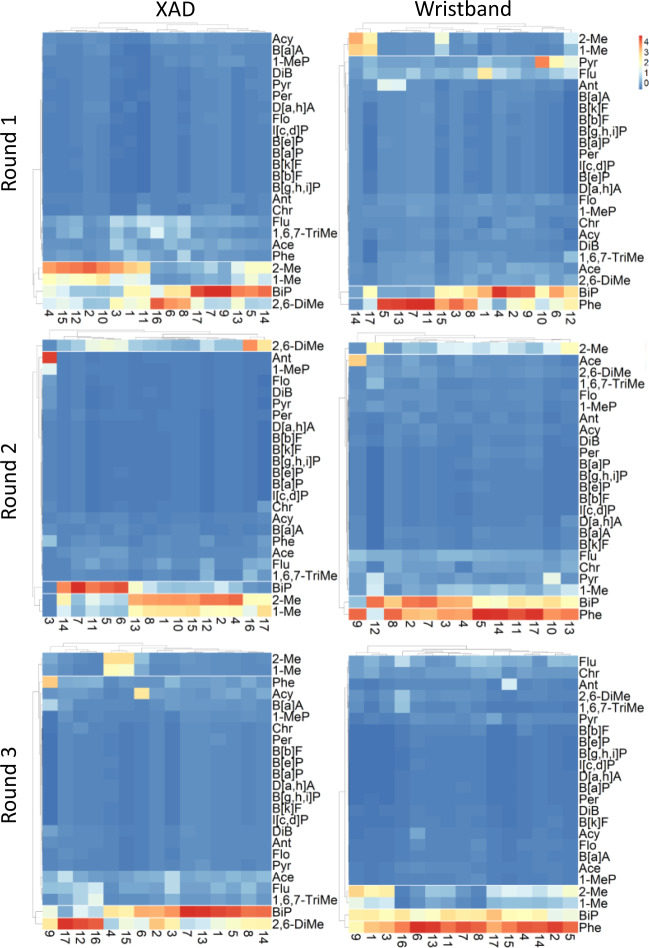
Heatmap of 24 EPA PAHs in individual’s XADs, and WBs grouped by sampling round. Acenaphthylene (Acy), acenaphthene (Ace), fluorene (Flu), phenanthrene (Phe), anthracene (Ant), fluoranthene (Flo), pyrene (Pyr), benzo(a)anthracene (B[a]A), chrysene (Chr), benzo(k)fluoranthene (B[k]F), benzo(a)pyrene (B[a]P), dibenz(a,h)anthracene (D[a,h]A), benzo(b)fluoranthene (B[b]F), benzo(e)pyrene (B[e]P), indeno[1,2,3-c,d]pyrene (I[c,d]P), benzo(g,h,i)perylene (B[g,h,i]P), 1-methylnaphthalene (1-Me), 2-methylnaphthalene (2-Me), biphenyl (BiP), 2,6-dimethylnaphthalene (2,6-DiMe), 1,6,7-trimethylnaphthalene (1,6,7-TriMe), 1-methylphenanthrene (1-MeP), dibenzothiophene (DiB), and perylene (Per).

To identify main PAH profiles for XAD and WBs, we obtained heatmaps using all the 24 PAH measured (Fig. 3). Results showed that biphenyl, 1-methylnaphthalene, and 2-methylnaphthalene concentrations are high when compared to other PAHs and this pattern is similar in the WB and XAD profiles. In addition, concentrations of 2,6-dimethylnaphthalene and phenanthrene are high in XAD and WB, respectively.

### Partition coefficient passive sampler to air

In this study, we placed the wristbands on the backpack strap to avoid dermal–wristband and other confounding interactions and calculate wristband to air partition coefficients under our deployment conditions. Partition coefficients were calculated based on the following equation that assumes passive sampler uptake is at equilibrium [22, 27].

$$K_{sa} = \frac{{C_{wb}}}{{C_{{\mathrm{air}}}}}$$

where *K*_*sa*_ is the passive sampler–air partition coefficient (L/kg), *C*_*wb*_ is the concentration measured in the wristband (ng of PAH in WB/mass of WB in kg), and *C*_air_ is the concentration in air (ng of PAH/L of air sampled) obtained from active sampling. We calculated the concentration in air using three active sampling concentrations: (ng of PAH in XAD/L of air sampled), (ng of PAH in filter/L of air sampled), and (ng of PAH in XAD plus filter/L of air sampled). Results shown in Fig. S4 showed that PAHs with octanol–air partitions coefficient smaller than log(*K*_*oa*_) = 8 follow a positive relationship with sampler–air partition coefficient.

**Fig. 4:**
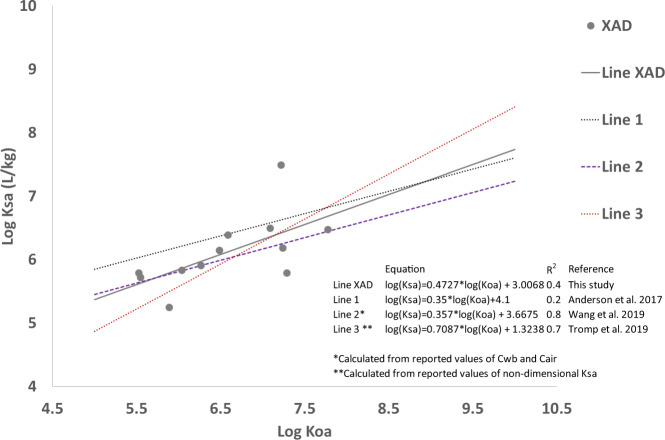
Partition coefficients relationship Log Koa vs Log Ksa. Ksa was calculated by dividing the concentration in WB by Concentration in air. Concentration in air was calculated using only XAD concentrations. Other lines are plotted based on published information.

Following *K*_*sa*_ calculations, and assuming passive sampler uptake at equilibrium for PAHs with log(*K*_*oa*_) values smaller than 8, we obtained linear equations that best described the relationship between median values of log(*K*_*sa*_) and log(*K*_*oa*_). We obtained three lines describing the relationship (Fig. S5), and observed that the relationships were different depending on the media used for the calculation of the concentration in air. We selected the linear relationship obtained from XAD values in this study to compare it with linear equations reported or calculated from previously published studies [22, 23, 25], where the concentration of air was obtained using either XADs, polyurethane foam (PUF), or both (Fig. 4). XAD values were selected because our analysis distinguished particle-bound PAHs absorbed in filters from vapor phase PAHs absorbed in XAD. Similarly, WB matrices were rinsed prior to extraction, which removed surface particles. In our study, evidence of sampler uptake at equilibrium was not obtained; however, the similarity between different linear equations suggests that PAHs with low Koa may reach equilibrium in 24 h.

## Discussion

Pregnancy represents a period of vulnerability for fetal exposure to PAHs and adverse health outcomes in childhood. Numerous cohort studies reveal adverse effects in children related to maternal airborne exposure, including birth defects, preterm birth, decreased birthweight, adverse cognitive development and asthma susceptibility [5–9, 11–14, 28]. In South Texas, specifically Hidalgo County, rates of prematurity and childhood asthma are elevated in comparison to state-wide data [17, 18]. Thus, environmental factors relevant to maternal and child health warrant investigation. A prior study in nearby Brownsville, Texas indicates that pregnant women were regularly expose to multiple PAHs, albeit at comparatively low concentrations, with levels in cord blood generally exceeding levels in maternal blood [29]. In this study conducted in the McAllen–Edinburg–Mission region, the most populated region in Hidalgo County, maternal PAH exposure was comparable to levels measured in participants from other U.S. cities. The median level of exposure for the sum of 16 PAHs measured via active sampling over 24 h was 5.54 ng/m^3^ (filters) and 43.82 ng/m^3^ (XADs). This is similar to levels reported in other U.S. populations. For instance, in a maternal cohort living in Fresno, California, investigators utilized a spatio-temporal model to assign daily estimated exposure to PAHs with 4, 5, or 6 rings [30]. In that population, the median PAH exposure during pregnancy was predicted to be 3.6 ng/m^3^, close to our filter values that capture higher molecular weight PAHs (i.e., 4, 5, or 6 rings). In New York City, Perera et al. [31] reported a median level of 2.273 ng/m^3^ from the sum of eight PAHs measured using an active sampling device with a quartz microfiber filter and a PUF cartridge over a 2-day period [31]. Following a similar sampling approach, Jedrychowski et al. reported a higher median level of 24.93 ng/m^3^ among pregnant women living in Krakow, a highly polluted city in Poland [11]. Thus, our active sampling data regarding 16 priority PAHs are comparable to levels measured in cohorts in other U.S. cities. In addition, we measured eight volatile PAHs using XAD sorbents (active sampler) and silicone wristbands (passive sampler, which results are discussed in detail in the next section). The median level of exposure for the sum of these eight PAHs was 342.98 ng/m^3^ (XADs). In our initial characterization of PM_2.5_ levels in our South Texas study population, we concluded vehicle emissions contributed considerably to the exposure of several participants, but also cooking emissions from modern indoor stoves significantly contributed to individual’s PM_2.5_ mass concertation. Notably, participants in our study spent the majority of their time indoors. Moreover, nicotine levels in participant hair samples were negligible, indicating a lack of exposure to environmental tobacco smoke [16]. Since prior studies indicate linkages of parental PAH exposure at this level with adverse health outcomes in children, these data suggest legitimate environmental health concerns related to maternal and child health. Further investigation on the associated health effects and preventive interventions are needed.

Our participant profiles were unique to each individual and sampling period. The high degree of variability between individuals and sampling days reflects natural variation in exposure. Indeed, we observed high variability in overall PM_2.5_ levels between participants and between sampling days [16]. We observed some statistically significant differences for specific participants over three different rounds. This variation reflects varying spatial and temporal patterns even though our participants were sampled over a relatively short (6-week) period, all within the 3rd trimester of pregnancy. It is well-documented that heterogeneity in study outcomes, e.g., preterm birth and birthweight, in developmental exposure studies is largely driven by the method of exposure assessment employed [32–34]. For developmental PM_2.5_ exposure, epidemiological studies routinely use ground-based monitoring or models to estimate PM_2.5_ exposure across pregnancy, and numerous studies report the method of exposure assessment heavily influences effect estimates related to birth outcomes. Similar to accurately characterizing PM_2.5_ exposure, it is critical to accurately characterize PAH exposure across pregnancy. This is particularly important based on the known transplacental transfer of PAHs [29, 35, 36] and reportedly heightened impact of in utero PAH exposure on infant birthweight reduction in comparison to PM_2.5_ exposure [15]. While personal monitoring using active sampling is largely prohibitive across pregnancy, passive sampling using silicone wristbands offers the opportunity to gain spatially refined chemical exposure data over longer durations. Notably, this technique affords investigators the ability to distinguish between inhaled versus dietary and dermal PAH sources, unlike urine or blood biomarkers, which reflect all routes of exposure.

Comparison of PAHs detected between different media (filters, XAD, and wristband) revealed interesting results. Assuming that filters absorb particle-bound PAHs while XAD and WB matrices absorb vapor phase PAHs, our results indicate that acenaphthylene, fluorene, and phenanthrene occurred in both the particle-bound and vapor phase. This assumption is appropriate since our methodology incorporated rinsing of WBs to remove particles prior analysis as well as collecting and analyzing two distinct matrices (filter and XAD) from the active sampler. Artifacts in active air sampling have been reported as potential concerns [37–39]. For example, gas-phase PAH absorbed on the filter or breakthrough of particle-bound PAH from the filter to the XAD could lead to miss-quantification of the gas/particle distribution. Testing for artifacts is out of the scope of this paper; however, the authors recognize that this is an understudied issue especially for PEMs. Statistically significant differences of measurements grouped by round showed high variation in PAH concentrations. These results indicate that chemical properties, e.g., particle-bound phase molecular weight and volatility, influence levels of individual PAH compounds across different sample matrices (filter, XAD, or silicone wristband). High variation among participants’ filters between the three rounds and low variation in participants’ profiles for XAD and WB was also observed. Our previous results for individual mobility, average daily PM_2.5_ concentrations, and percent contribution from various microenvironments show intra-individual variation across sampling days, which could be comparable to filter observations in the present study.

In regards to PAH levels measured using silicone wristbands, we placed the wristbands on the backpack strap capturing only potential inhalation exposure. In addition, the deployment time was shorter in our study compared to other studies (1 compared to 4 to 7 days). Then, results from our study are not comparable to other studies where wristbands were worn by participants for longer periods. However, we observed some key similarities. For instance, in a number of studies that quantified personal exposure to PAHs using wristbands, phenanthrene was the most abundant PAH quantified [19, 26, 25, 40–43] similar to our findings that phenanthrene was the predominant PAH in WB samples. Phenanthrene is an abundant component of indoor air pollution resulting from combustion during cooking and heating [29]. Indeed, cooking emissions were found to be a significant PM_2.5_ source for many of our study participants [16]. The second PAH most abundant in the wristbands of our study was fluorene. Similarly, fluorene was among the most abundant PAHs quantified in other studies conducted in different settings, namely: a rural area with active natural gas extraction operations in Ohio [40]; pregnant women living in New York City [19]; a Native American community located in Washington State [42]; and Appalachian mining communities [43]. While it is difficult to compare PAH levels across studies due to different PAH compounds quantified, sampling duration, and units reported (ng/wristband or ng/g) in general, our results suggest similar phenanthrene and fluorene capture by silicone wristbands across different deployment settings. In addition, silicone wristbands and XAD sorbents bound 1-methynaphthalyne, 2-methylnaphthalene, biphenyl at high levels following similar patterns of detection. This reflects the ability of silicone wristbands to bind smaller molecular weight, semivolatile PAHs similar to XAD resin. While the toxicity of lower molecular weight PAHs is considered orders of magnitude lower than high molecular weight PAHs [44], exposure during this window of vulnerability may elicit toxic effects. Furthermore, epidemiological evidence suggests that in particular low molecular weight (two-four rings), PAHs may be associated with nonmalignant pulmonary effects [45].

Previous studies report different approaches for calibrating passive samplers. A first approach is to characterize passive sampler–air partitioning behavior by means of empirical free energy relationships or models that account for sampler physical properties [24, 46]. A second approach is the use specialized exposure chambers to estimate gas-phase uptake rate, mass transfer, and sampler–air partition coefficients under controlled conditions of constant chemical in air, wind, temperature, and humidity [23, 47]. Another approach involves co-deployment of traditional active sampling systems and passive samplers placed in fixed indoor or outdoor locations to obtain gas-phase uptake and partition coefficients [27, 48, 49]. There are only a handful of studies with paired deployment of personal passive sampler (wristband, lapel, brooch) and personal active sampler (air monitoring backpacks) that capture individual variation in indoor and outdoor exposure [22, 25, 50]. The paired deployment approach was incorporated in our study design. We observed that partition coefficients calculated were similar to previous calculations from other studies conducted for PAHs with the same material of our wristbands [21, 24, 26]. The similarity between different linear equations suggests that PAHs with low Koa may reach equilibrium in 24 h. Anderson et al. [22] reported that equilibrium was attained at 24 h for PAHs with low molecular weight. One difference in our study versus that of Anderson et al. [22] is the placement of the band onto the backpack monitor versus participant wrist. We chose this option to control for other variables that could affect chemical uptake in the wristband, for example, use of personal care products or dermal–wristband interactions. For calibration of wristbands under variable indoor–outdoor exposure conditions, other studies on integrated dermal and inhalation exposure [25, 48] and our results provide evidence that more studies are needed. First, two types of wristband configurations could be incorporated during paired deployment: worn by participants to obtain dermal and inhalation exposure; and attached to personal monitoring equipment to obtain air concentrations. This configuration would be necessary to calibrate wristbands under individual exposure variation. In addition, different deployment periods could aid to corroborate if equilibrium is attained under day-to-day exposure variation. In our study, silicone wristbands bound smaller molecular weight, semivolatile PAHs in a similar way as the XAD media. The heatmaps showed that biphenyl, 1-methylnaphthalene, and 2-methylnaphthalene levels had a similar pattern of detection between XAD and WB, indicating that silicone wristbands may yield quantitative data for short periods of exposure if PAHs of low molecular weight are considered. Finally, we followed the original analytical methods by O’Connell et al. [26], including rinsing the wristbands with deionized water and isopropanol to dry prior to extraction, which may have unintentionally removed larger particulates. This methodology was appropriate since our study design incorporated analysis to distinguish particle-bound PAHs absorbed in filters from active samplers and vapor phase PAHs absorbed in XAD and WB matrices from active and passive samplers. Further studies could incorporate analytical methodologies designed to either remove and/or include PM-bound PAHs if the aim is to characterize those differences.

## Conclusions

We measured maternal PAH exposure in a South Texas population using active and passive sampling techniques. Results obtained from traditional active sampling indicated that PAHs are comparable to levels measured in other U.S. cities. PAH exposure and adverse health outcomes in children have been reported for similar PAH levels detected in our study, which justifies further investigation on the associated health effects and preventive interventions. Results comparing active with passive sampling, shows that biphenyl, 1-methylnaphthalene, and 2-methylnaphthalene were quantified in similar profiles in silicone wristbands and XAD sorbents, reflecting the ability of the passive sampler to bind smaller molecular weight, semivolatile PAHs in a similar pattern as active sampling. The application of inexpensive and convenient devices, such as silicone wristbands, to yield quantitative air quality data for continuous personal monitoring is appealing especially for windows of susceptibility such as pregnancy. Wristbands have the potential to yield concentrations of organic compounds in air and partition coefficients may be used to obtain those concentrations with a degree of uncertainty. More work regarding wristband calibration under field conditions is needed to characterize the level of uncertainty and uptake capacity.

## Supplementary information

Supplementary Information
