# Supplementary material for: Maternal exposure to polycyclic aromatic hydrocarbons in South Texas, evaluation of silicone wristbands as personal passive samplers
Source: J Expo Sci Environ Epidemiol. 2021 Jun 15;32(2):280–8. doi: 10.1038/s41370-021-00348-y (PMC8920889; doi:10.1038/s41370-021-00348-y)
Supplement: Supplementary file 1 — Supplementary Information [file 41370_2021_348_MOESM1_ESM.docx]

**Supplemental Information**

**Figure S1**

**
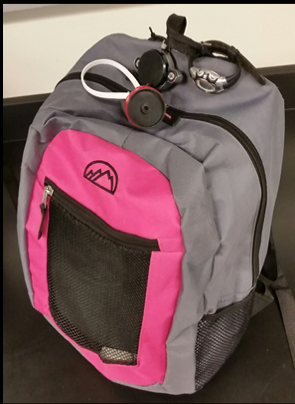
**

**S1.** Picture showing the backpack carried by participants containing active and passive samplers. A personal environmental monitor (PEM, MSP Inc.) was used as a single-stage impactor PM_2.5_ inlet (shown in back). Shown in front of the PEM, a pre-cleaned silicone wristband was attached near the inlet of the active air sampler. A watch was placed on the outside of the backpack to record absolute time. Details of equipment are descried in the methods section.

**Figure S2**

**S2.** Heat map of 16 EPA PAHs in participant’s Filters, XAD, and WBs grouped by sampling round. Abbreviations: acenaphthylene (Acy), acenaphthene (Ace), fluorene (Flu), phenanthrene (Phe), anthracene (Ant), fluoranthene (Flo), pyrene (Pyr), benzo(a)anthracene (B[a]A), chrysene (Chr), benzo(k)fluoranthene (B[k]F), benzo(a)pyrene (B[a]P), dibenz(a,h)anthracene (D[a,h]A), benzo(b)fluoranthene (B[b]F), benzo(e)pyrene (B[e]P), indeno[1,2,3-c,d]pyrene (I[c,d]P), and benzo(g,h,i)perylene (B[g,h,i]P), 1-methylnaphthalene (1-Me), 2-methylnaphthalene (2-Me), biphenyl (BiP), 2,6-dimethylnaphthalene (2,6-DiMe), 1,6,7-trimethylnaphthalene (1,6,7-TriMe), 1-methylphenanthrene (1-MeP), dibenzothiophene (DiB), and perylene (Per). The color of the heat map for Filter Round 2 seems uniform; this is because PAH concentrations of participant 8 are one order of magnitude larger than other participants’ concentrations.

**Figure S3**

**S3.** Heat map of additional 8 PAHs in participant’s XAD and WBs grouped by sampling round. Abbreviations: acenaphthylene (Acy), acenaphthene (Ace), fluorene (Flu), phenanthrene (Phe), anthracene (Ant), fluoranthene (Flo), pyrene (Pyr), benzo(a)anthracene (B[a]A), chrysene (Chr), benzo(k)fluoranthene (B[k]F), benzo(a)pyrene (B[a]P), dibenz(a,h)anthracene (D[a,h]A), benzo(b)fluoranthene (B[b]F), benzo(e)pyrene (B[e]P), indeno[1,2,3-c,d]pyrene (I[c,d]P), and benzo(g,h,i)perylene (B[g,h,i]P), 1-methylnaphthalene (1-Me), 2-methylnaphthalene (2-Me), biphenyl (BiP), 2,6-dimethylnaphthalene (2,6-DiMe), 1,6,7-trimethylnaphthalene (1,6,7-TriMe), 1-methylphenanthrene (1-MeP), dibenzothiophene (DiB), and perylene (Per).

**Figure S4**

**S4.** Partition coefficients relationship Log Koa vs Log Ksa. Ksa was calculated by dividing the concentration in WB by Concentration in air. Concentration in air was calculated using only Filter concentrations (regression line y = 0.34x + 5.26 R² = 0.04), only XAD concentrations (regression line y = 0.5x + 2.87 R² = 0.19) or Filter + XAD concentrations (regression line y = 0.67x + 1.80 R² = 0.32).

**Figure S5**

**
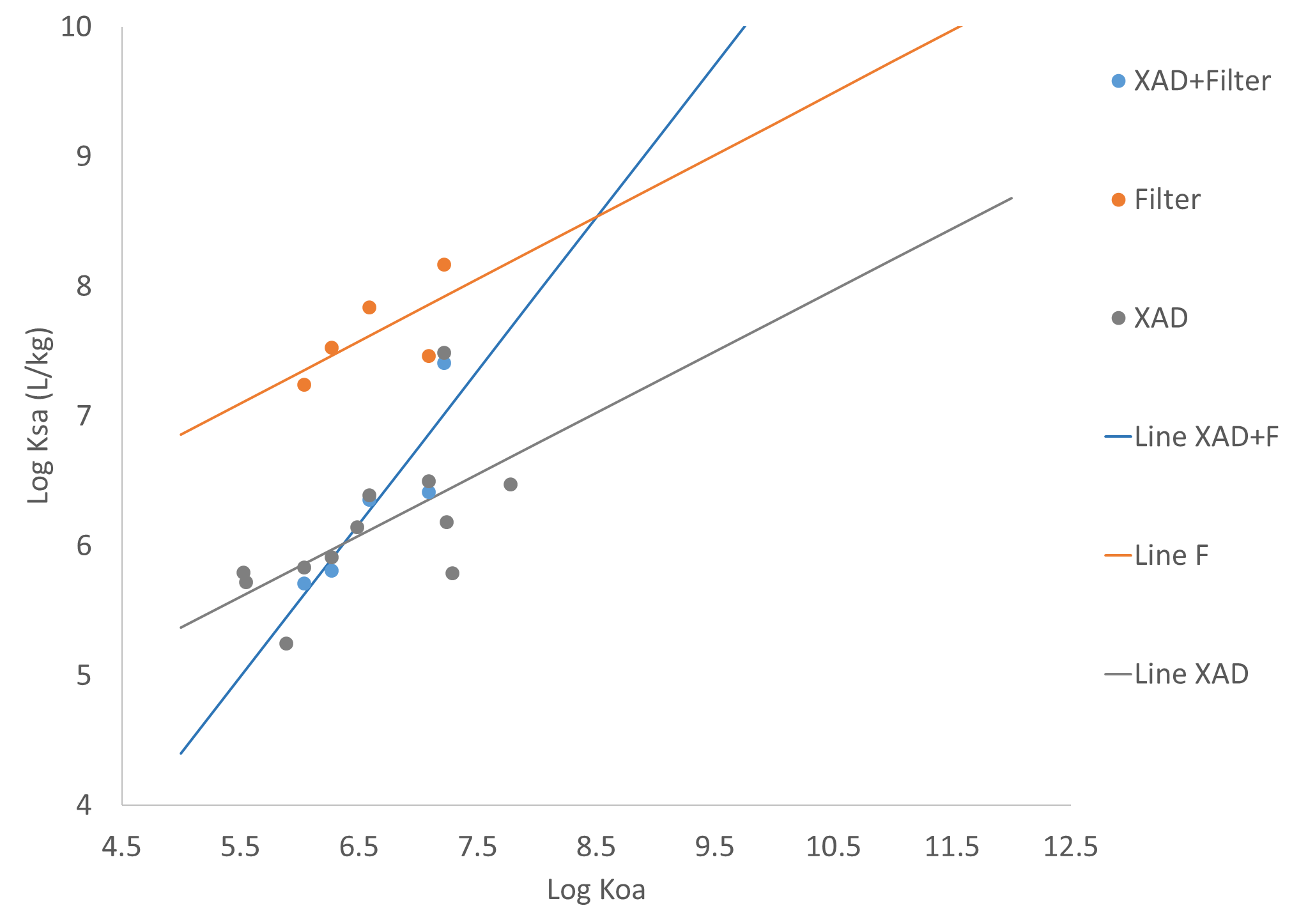
**

**S5.** Partition coefficients relationship Log Koa vs Log Ksa. Ksa was calculated by dividing the concentration in WB by Concentration in air. Concentration in air was calculated using only XAD concentrations, only Filter concentrations or Filter + XAD concentrations.

**Table S1.** Statistically significant correlation between temperature and PAH in each media (p-value is below 0.05)

|  | **XAD** | **WB** | **Filter** |
| --- | --- | --- | --- |
| Biphenyl |  |  |  |
| Acenaphthylene |  |  |  |
| Acenaphthene | 0.38 |  |  |
| Fluorene |  |  |  |
| Phenanthrene |  |  |  |
| Anthracene |  |  |  |
| Dibenzothiophene |  |  |  |
| Fluoranthene |  |  |  |
| Pyrene |  |  |  |
| Benzo(a)anthracene |  |  |  |
| Chrysene |  |  |  |
| Benzo(b)fluoranthene |  |  |  |
| Benzo(k)fluoranthene |  |  |  |
| Benzo(e)pyrene |  |  |  |
| Benzo(a)pyrene |  |  |  |
| Perylene |  |  |  |
| Indeno[1,2,3-cd]pyrene |  | 0.33 |  |
| Dibenz(a,h)anthracene | -0.41 |  |  |
| Benzo(g,h,i)perylene |  |  |  |
| 2-Methylnaphthalene |  |  |  |
| 1-Methylnaphthalene |  |  |  |
| 2,6-Dimethylnaphthalene | 0.55 |  |  |
| 1,6,7-Trimethylnaphthalene | 0.38 |  |  |
| 1-Methylphenanthrene |  |  |  |

**Table S2.** Statistically significant differences evaluated for the concentration profile of each PAH grouped by round (ssd means that Wilcoxon signed-rank p-value is below 0.05)

|  | **XAD** | | | **WB** | | | **Filter** | | |
| --- | --- | --- | --- | --- | --- | --- | --- | --- | --- |
|  | **R1-R2** | **R1-R3** | **R2-R3** | **R1-R2** | **R1-R3** | **R2-R3** | **R1-R2** | **R1-R3** | **R2-R3** |
| Biphenyl |  |  |  |  |  |  |  |  |  |
| Acenaphthylene |  |  |  |  |  |  |  |  |  |
| Acenaphthene | ssd |  | ssd |  | ssd |  |  | ssd |  |
| Fluorene | ssd | ssd |  |  |  |  |  |  |  |
| Phenanthrene | ssd | ssd |  |  |  |  |  |  |  |
| Anthracene |  | ssd |  |  | ssd | ssd |  |  |  |
| Dibenzothiophene | ssd |  |  |  |  |  |  |  |  |
| Fluoranthene |  |  |  |  | ssd |  |  |  |  |
| Pyrene | ssd | ssd |  |  | ssd | ssd |  |  |  |
| Benzo(a)anthracene |  |  |  | ssd | ssd |  |  |  |  |
| Chrysene |  | ssd | ssd | ssd | ssd | ssd |  | ssd |  |
| Benzo(b)fluoranthene |  |  |  |  | ssd | ssd |  |  |  |
| Benzo(k)fluoranthene |  |  |  | ssd | ssd |  |  | ssd | ssd |
| Benzo(e)pyrene |  | ssd |  |  | ssd | ssd |  |  |  |
| Benzo(a)pyrene |  |  |  |  |  |  |  |  |  |
| Perylene |  | ssd | ssd | ssd | ssd |  |  |  |  |
| Indeno[1,2,3-cd]pyrene |  | ssd | ssd |  | ssd | ssd |  |  |  |
| Dibenz(a,h)anthracene |  | ssd | ssd | ssd |  | ssd |  | ssd |  |
| Benzo(g,h,i)perylene |  |  |  |  |  |  |  |  |  |
| 2-Methylnaphthalene |  | ssd | ssd | ssd | ssd |  |  |  |  |
| 1-Methylnaphthalene |  | ssd | ssd | ssd | ssd |  |  |  |  |
| 2,6-Dimethylnaphthalene |  |  |  |  |  |  |  |  |  |
| 1,6,7-Trimethylnaphthalene |  |  |  |  |  |  |  |  |  |
| 1-Methylphenanthrene | ssd | ssd |  |  |  |  |  |  |  |

**Table S3.** Statistical significant differences evaluated for the PAH profiles of each participant grouped by round (ssd means that Wilcoxon signed-rank p-value is below 0.05)

|  | **XAD** | | | **WB** | | | **Filter** | | |
| --- | --- | --- | --- | --- | --- | --- | --- | --- | --- |
|  | **R1-R2** | **R1-R3** | **R2-R3** | **R1-R2** | **R1-R3** | **R2-R3** | **R1-R2** | **R1-R3** | **R2-R3** |
| 1 | ssd | ssd |  |  | | |  | ssd | ssd |
| 2 | ssd | ssd |  |  |  |  | ssd |  | ssd |
| 3 |  |  | ssd |  |  |  |  | | |
| 4 |  |  |  |  |  |  |  |  | ssd |
| 5 |  |  |  |  |  |  |  |  |  |
| 6 |  |  |  |  | | |  | ssd |  |
| 7 |  |  |  |  |  |  |  | ssd | ssd |
| 8 | ssd | ssd |  |  |  |  | ssd |  | ssd |
| 9 |  | | |  |  |  |  | | |
| 10 |  | | |  | | | ssd |  | ssd |
| 11 |  | | |  |  |  |  |  |  |
| 12 |  |  |  |  | | |  | | |
| 13 |  |  |  |  |  |  |  | ssd |  |
| 14 |  |  |  |  |  |  |  |  |  |
| 15 |  |  |  |  | | |  |  |  |
| 16 |  |  |  |  | | | ssd | ssd | ssd |
| 17 |  |  |  | ssd | ssd |  |  |  |  |
